# Toll-Like Receptor (TLR)-1/2 Triggering of Multiple Myeloma Cells Modulates Their Adhesion to Bone Marrow Stromal Cells and Enhances Bortezomib-Induced Apoptosis

**DOI:** 10.1371/journal.pone.0096608

**Published:** 2014-05-02

**Authors:** Jahangir Abdi, Tuna Mutis, Johan Garssen, Frank A. Redegeld

**Affiliations:** 1 Division of Pharmacology, Utrecht Institute for Pharmaceutical Sciences, Faculty of Science, Utrecht University, Utrecht, the Netherlands; 2 Department of Clinical Chemistry & Hematology, University Medical Center Utrecht, Utrecht, the Netherlands; University of Illinois at Chicago, United States of America

## Abstract

In multiple myeloma (MM), the malignant plasma cells usually localize to the bone marrow where they develop drug resistance due to adhesion to stromal cells and various environmental signals. Hence, modulation of this interaction is expected to influence drug sensitivity of MM cells. Toll-like receptor (TLR) ligands have displayed heterogeneous effects on B-cell malignancies and also on MM cells in a few recent studies, but effects on adhesion and drug sensitivity of myeloma cells in the context of bone marrow stromal cells (BMSCs) have never been investigated. In the present study, we explored the modulatory effects of TLR1/2 ligand (Pam3CSK4) on adhesion of human myeloma cells to BMSCs. It is shown that TLR1/2 triggering has opposite effects in different HMCLs on their adhesion to BMSCs. Fravel, L363, UM-6, UM-9 and U266 showed increased adhesion to BMSC in parallel with an increased surface expression of integrin molecules α4 and αVβ3. OPM-1, OPM-2 and NCI-H929 showed a dose-dependent decrease in adhesion upon TLR activation following a downregulation of β7 integrin expression. Importantly, TLR1/2 triggering increased cytotoxic and apoptotic effects of bortezomib in myeloma cells independent of the effect on stromal cell adhesion. Moreover, the apoptosis-enhancing effect of Pam3CSK4 paralleled induction of cleaved caspase-3 protein in FACS analysis suggesting a caspase-dependent mechanism. Our findings uncover a novel role of TLR activation in MM cells in the context of bone marrow microenvironment. Stimulation of TLR1/2 bypasses the protective shield of BMSCs and may be an interesting strategy to enhance drug sensitivity of multiple myeloma cells.

## Introduction

Adhesion of multiple myeloma (MM) cells to bone marrow stromal cells (BMSCs), mediated mostly by the integrin family of adhesion molecules, renders the tumor cells resistant against drugs and apoptotic stimuli, and contributes to other complications of the disease including osteolytic lesions and angiogenesis[1], [2], [3]. Several cytokines derived from both bone marrow stromal cells and MM cells have been indicated to maintain this interaction [4], [5], [6]. Toll-like receptors (TLRs) are a family of pathogen recognition receptors expressed mainly by the innate immune cells, but also by a variety of human cancer cells including those of B cell malignancies especially MM [7], [8], [9], [10], [11], [12]. TLR activation by microbial or endogenous ligands has been implicated in linking inflammation to cancer, with the transcription factor NFκB activation as the main establishing event [13], [14], [15], [16], [17], [18]. However, activation of NFκB in human myeloma cell lines (HMCLs) and primary MM cells has been explained partly by detection of some mutations in NFκB-controlled/related genes (mostly in alternative pathway) [19], [20], and are probably independent of TLR signaling which is normally through the canonical pathway [21], [22].

Possible contribution of TLRs to inflammation-related malignancy is indicated mostly by induction of pro-inflammatory cytokines in tumor environment [23], upregulation of cell adhesion molecules on cancer cells and their adhesion or migration following TLR triggering [12], [24], [25], [26]. Recent studies in cells of B lymphoid malignancies including MM also demonstrated that TLR triggering would result in both positive and negative outcomes, including induction of growth and proliferation, drug resistance, immune evasion and cell death. Nonetheless, the modulatory effect of TLR activation in MM cells on their adhesion to bone marrow microenvironment components including BMSCs has not been explored to date. Hence, regarding the fact that TLRs of MM cells may be activated in the inflammatory environment of bone marrow, possibly by microbial/endogenous ligands, we hypothesized that TLR triggering on MM cells might modulate their adhesion to BMSCs and subsequently modulate MM cells survival and drug resistance. In a recent study, we demonstrated that TLR1/2 activation either increased or decreased adhesion of human myeloma cells to fibronectin and modulated cytotoxicity of bortezomib in HMCLs [27]. In this study, we extend these previous observations and show using an *in vitro* adhesion system that TLR-1/2 triggering on MM cells by Pam3CSK4 modulated their interaction with BMSCs involving adhesion molecules of β1 integrin family. Furthermore, Pam3CSK4 treatment of HMCLs increased their apoptotic response to bortezomib in the context of BMSCs, which suggests that TLR1/2 triggering may be of therapeutic use to decrease cellular resistance to the cytotoxic action of chemotherapeutic agents.

## Materials and Methods

### Reagents and Antibodies

TLR-1/2 specific ligand, Pam3CSK4, was obtained from Invivogen (San Diego, CA, USA). Rat anti-human beta 7 integrin (clone FIB504, for both FACS and blocking), mouse anti-human αVβ3 integrin (CD51/CD61, clone 23C6, for both FACS and blocking), mouse anti-human VCAM-1 (CD106)-PE (clone STA), mouse anti-human CD49e (α5 integrin, clone P1D6)-PE, mouse anti-human CD49d (α4 integrin, clone 9F10)-PE, anti-mouse IgG-FITC, and mouse IgG2b, κ isotype control were all from eBioscience. Monoclonal rabbit anti-human MyD88 (clone D80F5) and anti-human cleaved caspase-3 (clone D3E9) were obtained from Cell Signaling Technology (Danvers, MA, USA). Mouse anti-human CD49d (clone HP2/1, for blocking) was from ABD Serotec (MorphoSys, Oxford, U.K). Alexa Fluor 488 rabbit anti-rat IgG (H+L) was purchased from Invitrogen. Anti-beta actin and horseradish peroxidase-conjugated goat anti-rabbit IgG were also from Santa Cruz Biotechnology, CA, USA. Bortezomib was obtained from LC Laboratories (Woburn, MA, USA) and dissolved in DMSO to make 100 mM stock. DMSO concentrations in all drug exposure tests never exceeded 0.05%. PMS (Phenazine methosulfate) and XTT were also supplied by Sigma-Aldrich.

### Cell Lines and Cell Culture

The HMCLs, Fravel, L363, OPM1, OPM2, U266, and NCI-H929, were obtained from American Type Culture Collection (Manassas, VA, USA). UM-6 and UM-9 had been established by the Department of Clinical Chemistry & Hematology, University Medical Center Utrecht, Utrecht, the Netherlands [28], [29]. UM6 is IL-6 dependent and others are IL-6 independent. All the cell lines were maintained in RPMI-1640 culture medium containing 2-mM L-glutamine supplemented with 5 or 10% fetal bovine serum and intermittently with antibiotics, in a 37°C incubator with 5% CO_2_. To UM6 cell line was added 5 ng/mL of recombinant human IL-6 (from eBioscience, San Diego, CA, USA). To NCI-H929 cell line medium were also added 1 mM sodium pyruvate and 50 µM 2-mercaptoethanol. Normal human bone marrow stromal cell line, HS-5, was obtained from American Type Culture Collection. This cell line was maintained in DMEM medium supplemented with 10% FBS and intermittently with antibiotics.

For isolating primary stromal cells, frozen vials of patient bone marrow samples were thawed, suspended in fresh warm DMEM medium and applied to Ficoll Hypaque density gradient centrifugation to possibly remove cellular debris and dead cells. The remaining fractions were suspended in DMEM medium and kept in a T-75 culture flask for a few hours in a 37°C incubator. Then the floating cells were gently aspirated and the adhered fraction was maintained in DMEM supplemented with 10% FBS, 100 IU/ml penicillin and 100 µg/ml streptomycin. The medium was refreshed twice per week to yield a confluent layer in 3–4 weeks. Confluent wells were passaged after detachment with trypsin-EDTA. The stromal cells at passage 1–3 were seeded in 12-well plates for survival experiments. Bone marrow samples were surplus material from bone marrow isolated for diagnostic procedures. All patients approved use of surplus material for scientific purposes by written informed consent. Use of surplus material has been discussed with and approved by the review board of the University Medical Center Utrecht. Due to the nature of the samples i.e. surplus sample remaining after diagnostic procedures, no formal approval number was needed and provided by the Ethics Committee.

### Cell Stimulation

To stimulate HMCLs, Pam3CSK4 was used in 1.0, 2.0 and 5.0 µg/ml concentrations. Before any treatment, cells were washed once with PBS, suspended in warm RPMI medium supplemented with 5% FBS. Incubation time in a 37°C incubator with 5% CO_2_ was 24 hours.

### Flow Cytometry

In FACS experiments, indirect or indirect staining was performed. Briefly, 10^5^ cells from indicated conditions were washed and suspended in FACS staining buffer (PBS+0.5% BSA+0.01% sodium azide). Cells were incubated with primary antibodies (β7 and αVβ3) followed by relevant fluorescent conjugated secondary antibodies. Direct staining method was also used for anti-VCAM-1 (CD106), anti-CD49d (α4) and anti-CD49e (α5) with fluorochrome-conjugated antibodies. Finally, the samples were washed, suspended in FACS buffer and analyzed with a FACSCantoII flow cytometer (BD Biosciences). Gated live cell populations were analyzed using Cell Quest or FACS Diva software.

### Fluorometric Adhesion Assay

Two to three days before adhesion experiments, 3×10^4^ cells of the bone marrow stromal cell line, HS-5, or MM primary BMSC were seeded on 96-well plates. Immediately before adhesion, the plates were washed twice with warm PBS. For adhesion analysis, 10^6^ HMCLs (treated or untreated) were harvested, washed twice in PBS buffer and suspended in 1 ml of room temperature RPMI medium without any additive. Cell suspensions were labeled with Calcein-AM (1 µM) for 30 minutes at room temperature with gentle mixing after 15 min. To stop labeling, samples were treated with ice cold RPMI and spun twice at 4°C. One milliliter of RPMI plus 2% FBS at room temperature was added to all samples and 10^5^ cells were seeded on stromal cell coated 96-well plates and incubated at 37°C for 2 hours. At the end of incubation time, total and background fluorescence were measured with a plate reader (Mithras LB 940, Berthold Technologies, Germany). For measuring fluorescence of adhered cells, non-adhered cells were removed with three gentle washes with warm RPMI, 100 µL RPMI was added to each well and the fluorescence was measured. In some experiments to determine the adhesion molecules involved, anti-β7 (5 µg/ml), anti-αVβ3 and anti-α4 (10 µg/ml) antibodies were added to appropriate number of cells for 15 min at 4°C, the cells were then washed once with cold RPMI to remove free antibody molecules and then added to coated plates. For background readings, fluorescence of the wells containing cells adhered only to BSA was considered. The following formula was used to calculate percentage of adhesion: (Fluorescence reading of adhered cells-background reading)×100/(Total fluorescence reading-spontaneous reading).

### Cell Survival: Drug Cytotoxicity Assay

Drug sensitivity measurements were performed using modification of an *acute exposure* approach as described previously [30]. HMCLs were first stimulated with 2 µg/ml (OPM-1, OPM-2, and NCI-H929) or 5 µg/ml (L363 and U266) Pam3CSK4 for 24 hours. Cells were washed twice with PBS and 5×10^4^ cells were treated with indicated concentrations of bortezomib in RPMI+5%FBS in separate 96-well round bottom plates for one hour at a 37°C incubator, with gentle shaking after 30 minutes. Cells were then washed with warm RPMI, resuspended in drug-free RPMI+FBS and transferred completely to the 96-well flat bottom plates pre-coated with HS-5. These plates were further incubated for 2–3 days. At the last 4 hours of incubation, 25 µl from XTT reagent which already contained PMS was added and incubation continued. Finally, the absorbance of each well was measured using a plate reader. The percent survival of cells was calculated by using non-linear regression. In each plate run, wells for solvent control (medium+cells+DMSO, for assay validity and also as 100% viability), blank (medium+DMSO), and growth control (medium+cells, for quality control) were also included. The readings of the blank wells were subtracted from those of all test samples.

### Cell Survival: Annexin-V/PI and Cleaved Caspase-3 Apoptosis Assay

Five hundred thousand cells from each HMCL were incubated in the presence or absence of Pam3CSK4 for 24 hours, washed and treated with 5 µM bortezomib in RPMI+FBS for one hour (*acute exposure).* Conditions without drug treatment were also included. Cells were then washed, resuspended in drug-free RPMI containing FBS and added to 12-well plates pre-coated with 1×10^5^ cells from HS-5cell line or patient BMSCs for 2 hours. Then unattached cells were removed and fresh medium containing protein was added and plates were incubated for 24–48 hours. In parallel, cells were also put in uncoated wells and treated as mentioned. Finally, cells were removed with cold 5 mM EDTA in PBS, washed and suspended in FACS buffer (cold PBS containing 1% BSA and 0.01% sodium azide). Samples were first stained with anti-CD138-APC for 45 minutes on ice, washed once with above FACS buffer and once with binding buffer (eBioscience). The cell pellets were then suspended in 200 µl binding buffer containing 5 µl FITC-conjugated annexin-V and incubated for 10 minutes at room temperature. After washing with binding buffer, 5 µl propidium iodide in 200 µl of this buffer was added to each well and samples were applied to FACS analysis in a BD FACS Canto II machine. The gate of CD138 positive cells was selected, and the percent-specific apoptosis was calculated using the following formula [27]: (Test-control)×100/(100-control). Test refers to the treatment with Pam3CSK4, bortezomib or Pam3CSK4+bortezomib, and control is the cells without any stimulation (baseline).For cleaved caspase-3 analysis, cells were first stained with andi-CD138 as above, fixed with a permeabilization/fixation buffer (eBioscience) for 30 minutes on ice, and then stained with anti-human cleaved caspase-3 for one hour at 4°C. At the next step, FITC-conjugated secondary antibody (anti-rabbit IgG) was added for 30 minutes on ice. After washing, samples were applied to FACS analysis as above.

### Statistical Analysis

We used unpaired t-test or ANOVA (one way) in GraphPad prism 5 software for statistical analysis, and the values with a p<0.05 were considered as significant.

## Results

### TLR-1/2 Triggering in HMCLs Modulates Surface Expression of Different Adhesion Molecules

**Figure 1:**
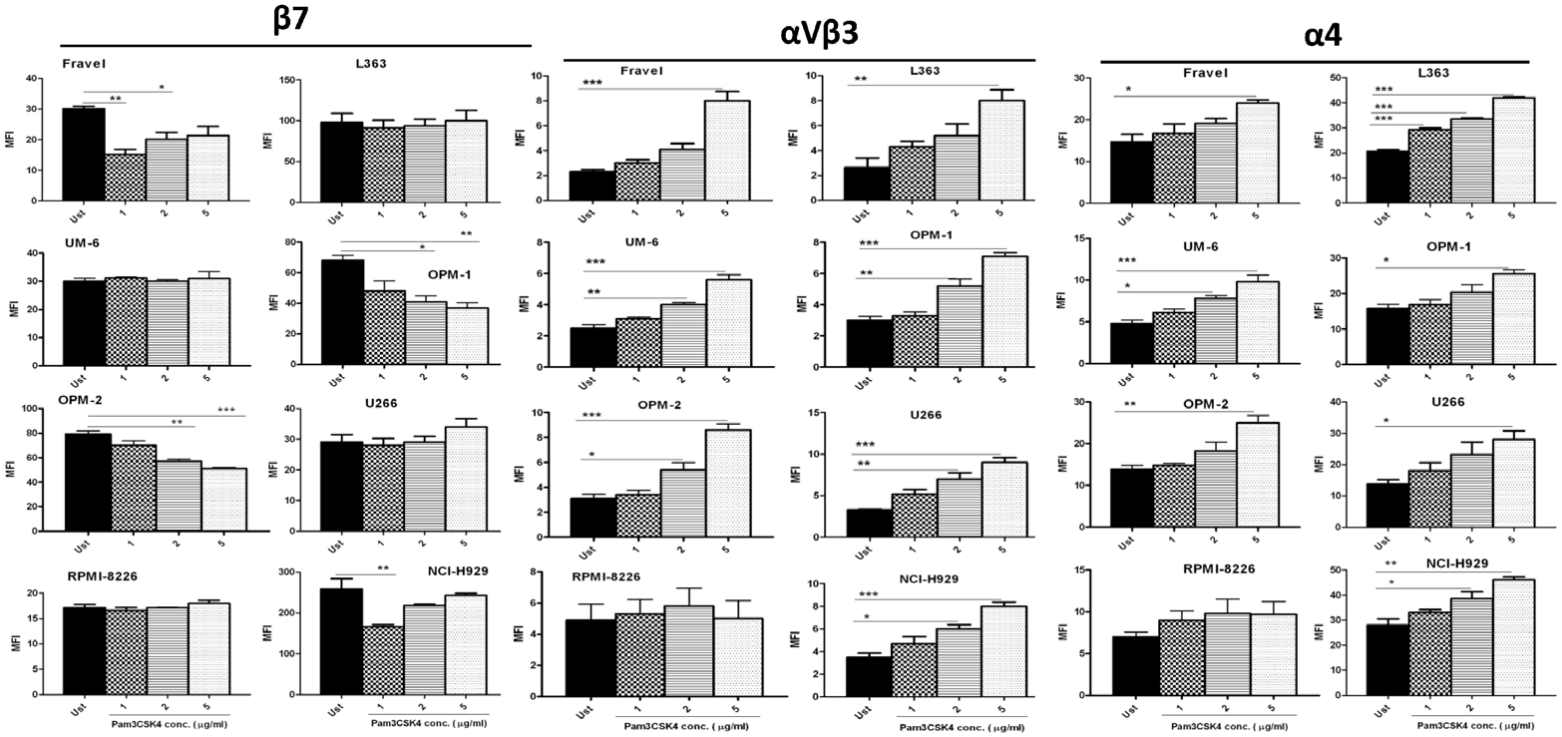
TLR-1/2 activation effects on expression of β7, αVβ3 and α4 integrins in HMCLs. HMCLs were stimulated with Pam3CSK4 for 24 hours and then applied to FACS analysis for expression of integrin molecules as explained in materials and methods. Pam3CSK4 downregulated β7 expression in Fravel, OPM-1, OPM-2, and NCI-H929 in a dose-dependent manner. No change in β7 expression was observed in any other cell line. Pam3CSK4 up-regulated αVβ3 and α4 expression dose-dependently in all HMCLs except RPMI-8226. The results are the statistical analyses of data in 3 separate experiments expressed as mean ± SEM, *P<0.05, **P<0.01, ***P<0.001. (Ust: Unstimulated).

**Figure 2:**
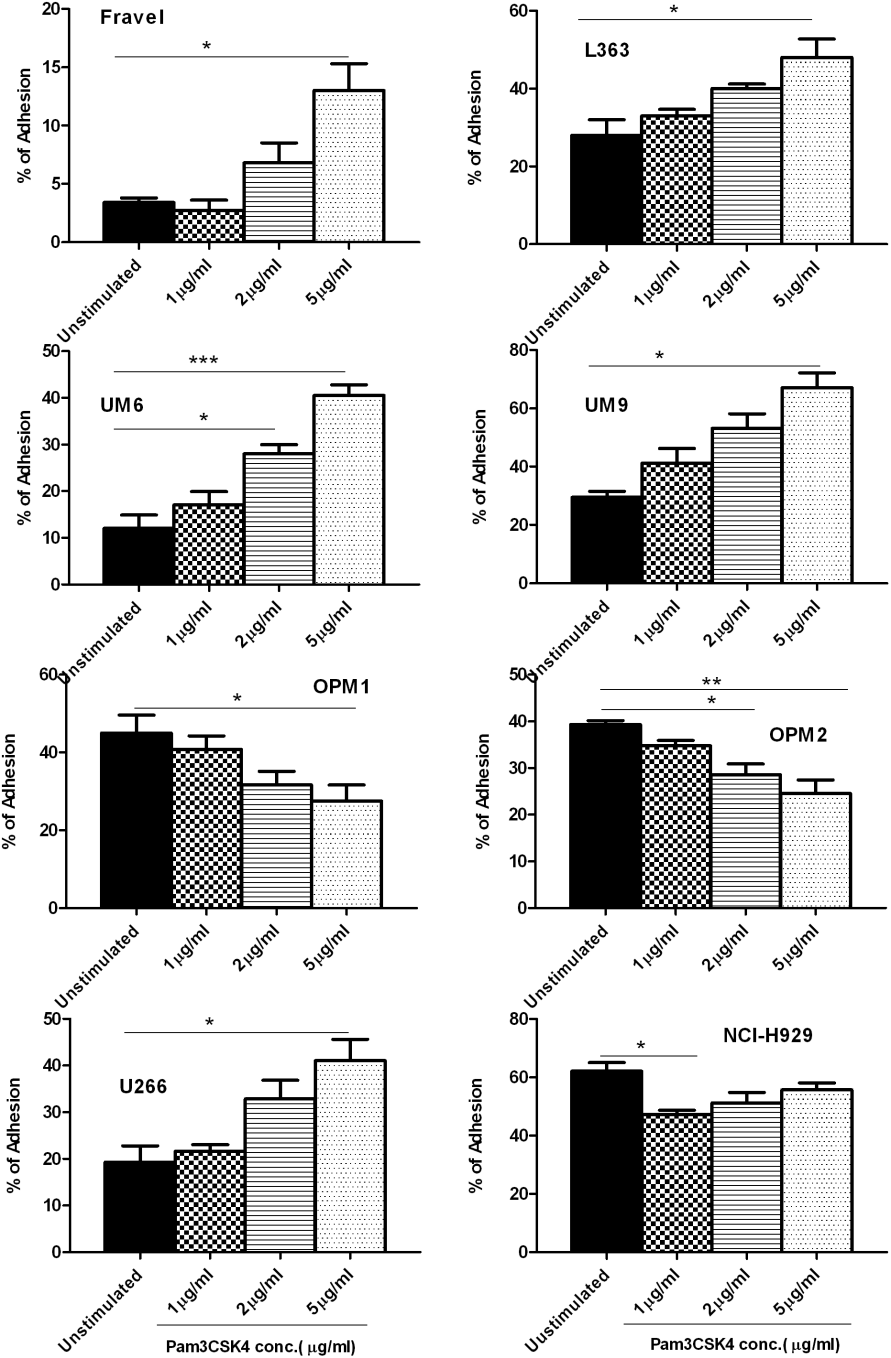
The effect of Pam3CSK4 on adhesion of HMCLs to BMSCs. HMCLs were stimulated with Pam3CSK4 for 24 hours and then exposed to HS5-coated wells for adhesion assay as explained in materials and methods. Pam3CSK4 decreased adhesion of OPM-1, OPM-2, and NCI-H929 cell lines to HS-5 in a dose-dependent manner. On the contrary, adhesion of L363, UM-6, UM-9 and U266 cell lines increased dose-dependently. The results are the statistical analyses of data in at least 3 separate experiments expressed as mean ± standard error of mean, *P<0.05, **P<0.01, ***P<0.001.

We first determined the effect of Pam3CSK4 on surface expression of integrin molecules β7, αVβ3, CD49d (α4) and CD49e (α5). Incubation with Pam3CSK4 resulted in down-regulation of β7 integrin on Fravel, OPM-1, OPM-2 and NCI-H929 cell lines in a dose-dependent manner (Fig. 1), with a pattern in OPM-1, OPM-2 and NCI-H929 cell lines closely matching their adhesion behavior (see Fig. 2). No change in β7 expression (or only minimal increase with the 5 µg/ml concentration) was observed in other cell lines. All cell lines displayed a dose-dependent increase in the expression of α4 and αVβ3 integrins, except RPMI-8226 which showed only small non-significant changes (Fig. 1). The α5 integrin was not detected on any of the cell lines except RPMI-8226 in which α5 expression was not affected by Pam3CSK4 treatment (data not shown).

### TLR-1 Triggering in HMCLs has Different Modulatory Effects on their Adhesion to BMSCs

We next investigated the effect of TLR1/2 ligand Pam3CSK4 on the interaction of MM cells with BMSCs, regarding the critical importance of this interaction in MM biology and pathogenesis. TLR1/2 activation modulated adhesion of HMCLs to BMSCs, yet with a heterogeneous pattern (Fig. 2). Fravel, L363, UM-6, UM-9 and U266 showed a dose-dependent increase in adhesion. Interestingly, Fravel and UM-6 showed quite low baseline adhesions (3.4% and 12%, respectively) which were highly increased with 5 µg/ml Pam3CSK4 (13% and 40%, respectively). OPM-1 and OPM-2 showed a dose-dependent decrease in adhesion. NCI-H929 showed maximal reduction of adhesion already at 1 µg/ml Pam3CSK4. The response of RPMI 8226 to TLR1/2 stimulation was inconsistent with respect to adhesion and therefore not further analyzed (data not shown).

### Modulation of HMCLs Adhesion to BMSCs following TLR-1 Activation Possibly Involves Different Integrin Molecules

**Figure 3:**
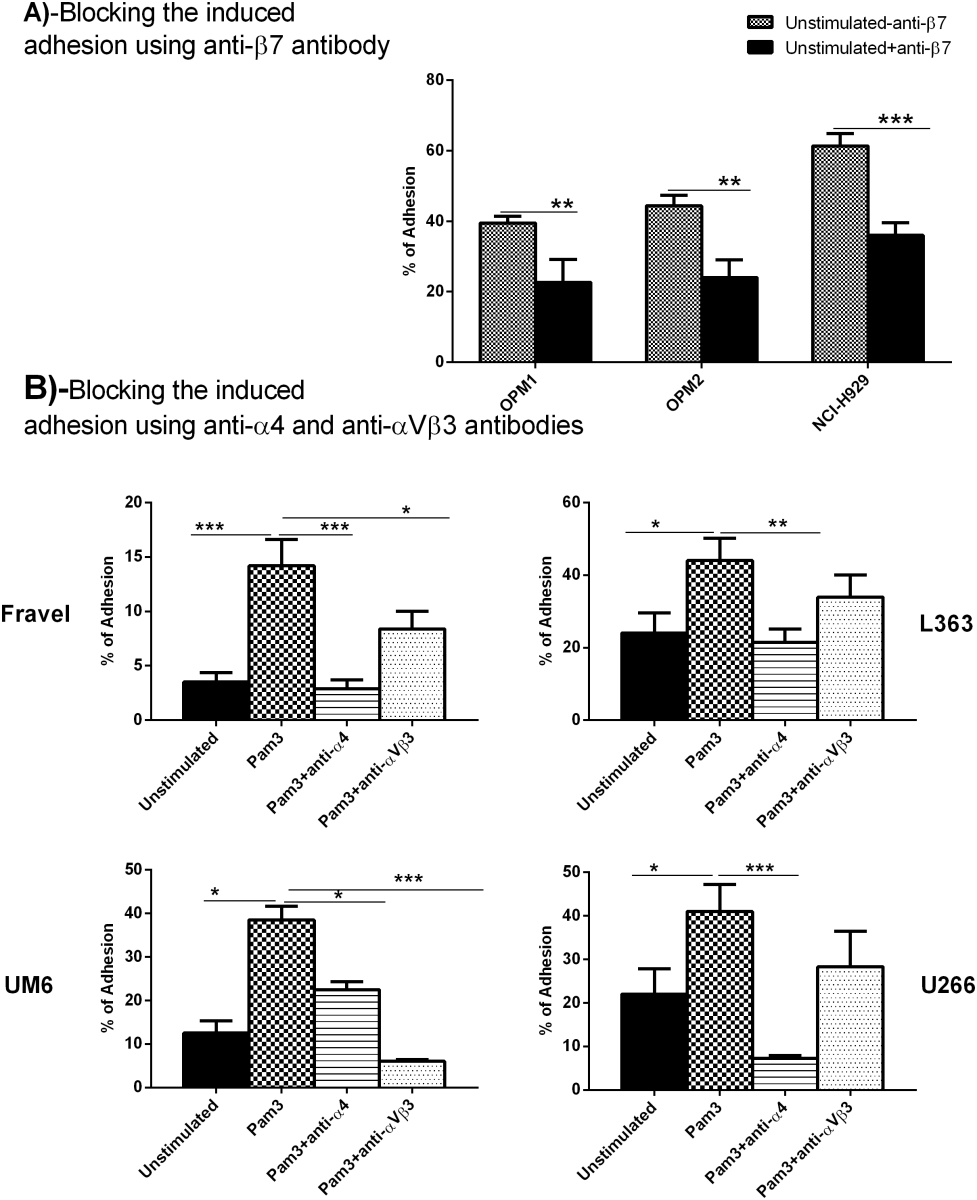
Blocking experiments with anti-β7, anti-α4 and anti-αVβ3 antibodies for adhesion to HS-5 stromal cells. Panel A, Blocking with anti-β7. HMCLs were stimulated with Pam3CSK4 for 24 hours and then treated with anti-β7 antibody before adhesion to HS5-coated wells. Panel B, Blocking experiments with anti-α4 and anti-αVβ3 antibodies for adhesion to HS-5. HMCLs were stimulated with Pam3CSK4 for 24 hours and then treated with anti-α4 and anti-αVβ3 antibodies before adhesion to HS5-coated wells. The results are the statistical analyses of data in 3 separate experiments expressed as mean ± SEM, *P<0.05, **P<0.01, ***P<0.001.

Based on the above findings, it seemed conceivable that β7 integrin could mediate down-regulatory and αVβ3 and α4 integrins up-regulatory effects of Pam3CSK4 on adhesion of HMCLs to BMSCs. To investigate this, we used anti-β7 antibody to block baseline adhesion to BMSCs in OPM-1, OPM-2, and NCI-H929. The anti-β7 antibody decreased baseline adhesion in these lines as much as 17%, 20%, and 25%, respectively (Fig. 3, panel A), indicating that β7 was involved in the adhesion of these HMCLs.

To investigate the involvement of αVβ3 and α4 integrins in adhesion, anti-αVβ3 and anti-α4 antibodies were used to inhibit adhesion of Fravel, L363, UM-6 and U266 cells at baseline or upon Pam3CSK4 stimulation (Fig. 3, panel B). The experiments indicated that Pam3CSK4-induced upregulation of adhesion was mediated by α4 and/or αVβ3 integrins. Adhesion of Fravel, L363 and U266 cell lines was shown to be mainly α4-mediated, as anti-α4 fully blocked the up-regulated level plus a large part of the baseline adhesion.

Blockade of αVβ3 integrin reduced Pam3CSK4-induced adhesion in Fravel, L363 and U266 cells but not as significantly as α4 blockade. This suggested that α4 integrin subunit was one of the main adhesion molecules engaged by above cell lines in adhesion to BMSCs. Adhesion of UM-6 was regulated by both α4 and αVβ3 integrins. These data confirmed the contribution of β7, α4, αVβ3 integrins to adhesion of HMCLs to stromal cells and suggested that there might be a heterogeneous response to TLR1/2 stimulation on their functional expression in HMCLs.

### TLR1 Activation in HMCLs Enhances Cytotoxic Effects of Bortezomib in the Context of Bone Marrow Stromal Cells

**Figure 4:**
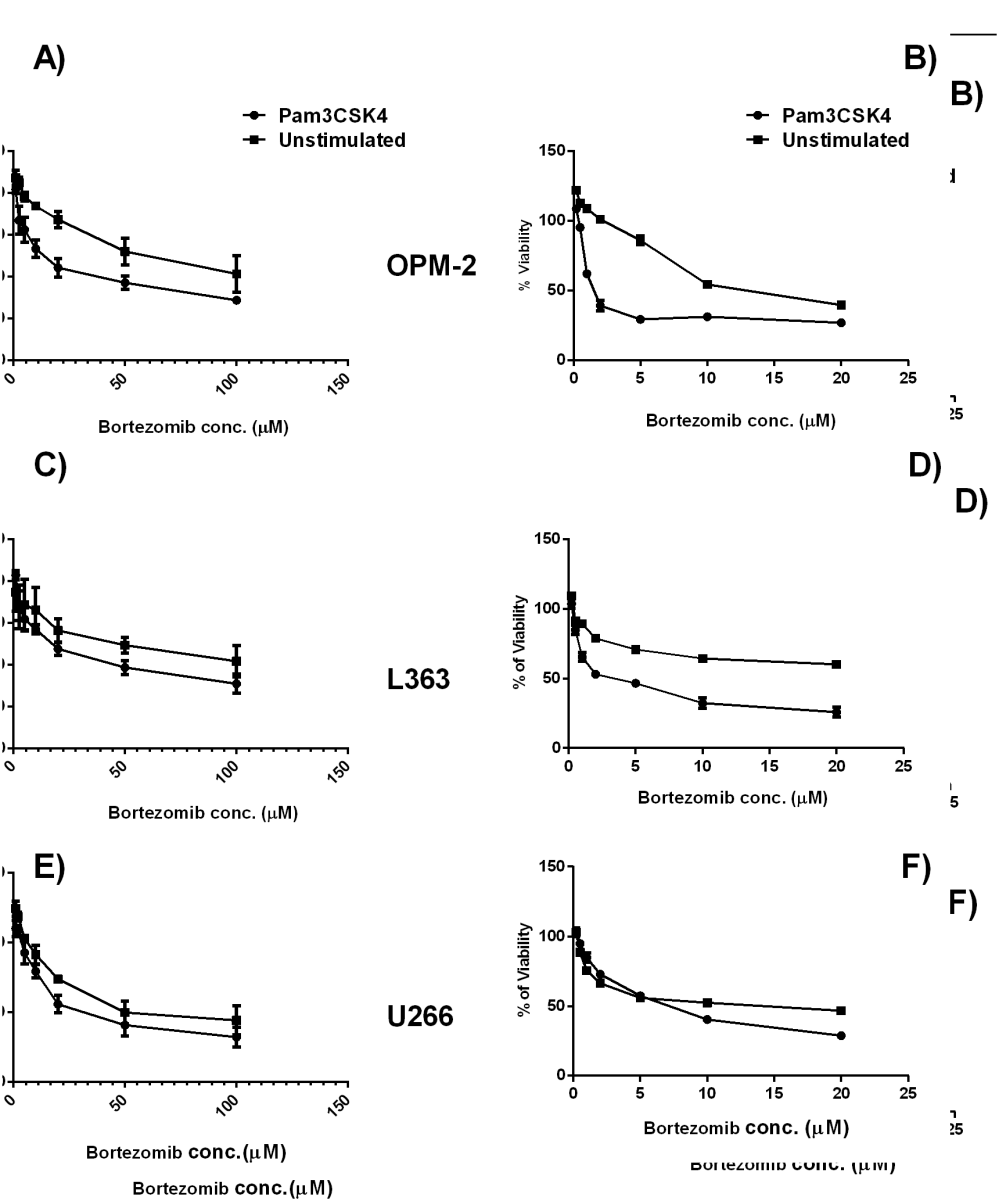
Pam3CSK4 increased sensitivity of HMCLs to bortezomib in the context of BMSCs. Myeloma cells were stimulated with Pam3CSK4 for 24 hours, and exposed to increasing drug concentrations in an acute manner. Panels A, C, E: HMCL adhered to BMSCs. Panels B, D, F: HMCLs in stroma-free conditions. OPM-2 (A, B), L363 (C, D) and U266 (E, F) HMCLs. Pam3CSK4 stimulated cells displayed a higher sensitivity to bortezomib in the presence or absence of stroma, however, the level of sensitivity was lower for HMCLs in BMSCs context than cells in the stroma-free condition. IC50s (µM of the drug): OPM-2: **stroma**, Pam3 (7.5 µM), unst (38.5 µM); **stroma-free**, Pam3 (1.9 µM), unst (15.6 µM); L363: **stroma**, Pam3 (12.8 µM), unst (41.1 µM); **stroma-free,** Pam3 (5.0 µM), unst (14.9 µM); U266: **stroma,** Pam3 (13.5 µM), unst (14.9 µM); **stroma-free,** Pam3 (5.6 µM), unst (11.2 µM).

Drug resistance of HMCLs can be greatly influenced by adhesion. In the next experiments, we investigated if the changed adhesion due to TLR1/2 stimulation would influence the drug sensitivity of HMCLs. L363 and U266 which showed upregulated and OPM-2 which showed downregulated adhesion following Pam3CSK4 treatment were selected for further investigation. As expected, IC50 of the HMCLs for bortezomib was higher when they adhered to stroma cells (Fig. 4). However, Pam3CSK4 treatment increased drug sensitivity of all HMCLs to bortezomib in the presence or absence of stromal cells suggesting that the Pam3CSK4-induced increases in bortezomib cytotoxicity were adhesion-independent.

### TLR1 Activation in HMCLs Enhances the Apoptotic Response to Bortezomib in the Context of Bone Marrow Stromal Cells

**Figure 5:**
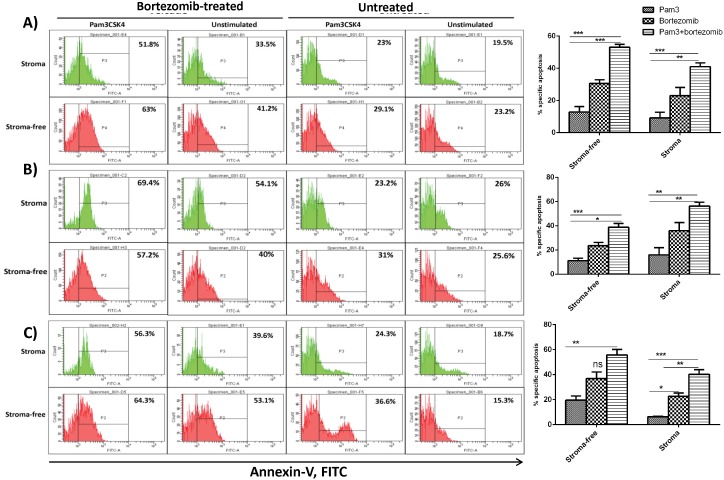
The apoptosis enhancing effect of Pam3CSK4 on HMCLs in the context of HS-5 stromal cells. Apoptosis in L363 (panel **A**), OPM-2 (panel **B**) and U266 (panel **C**) was determined by flow cytometric analysis of annexin-V binding. Percentage of apoptotic cells was calculated by selecting the gated CD138 positive cells. Left panels are one representative out of three separate experiments for each cell line and the right panels are statistical analyses of all experiments. HMCLs were stimulated for 24 hours with Pam3CSK4, adhered to HS-5 cells and then exposed to drug treatment as explained in materials and methods. The results are the statistical analyses of data in 3 separate experiments expressed as mean ± SEM, *P<0.05, **P<0.01, ***P<0.001.

**Figure 6:**
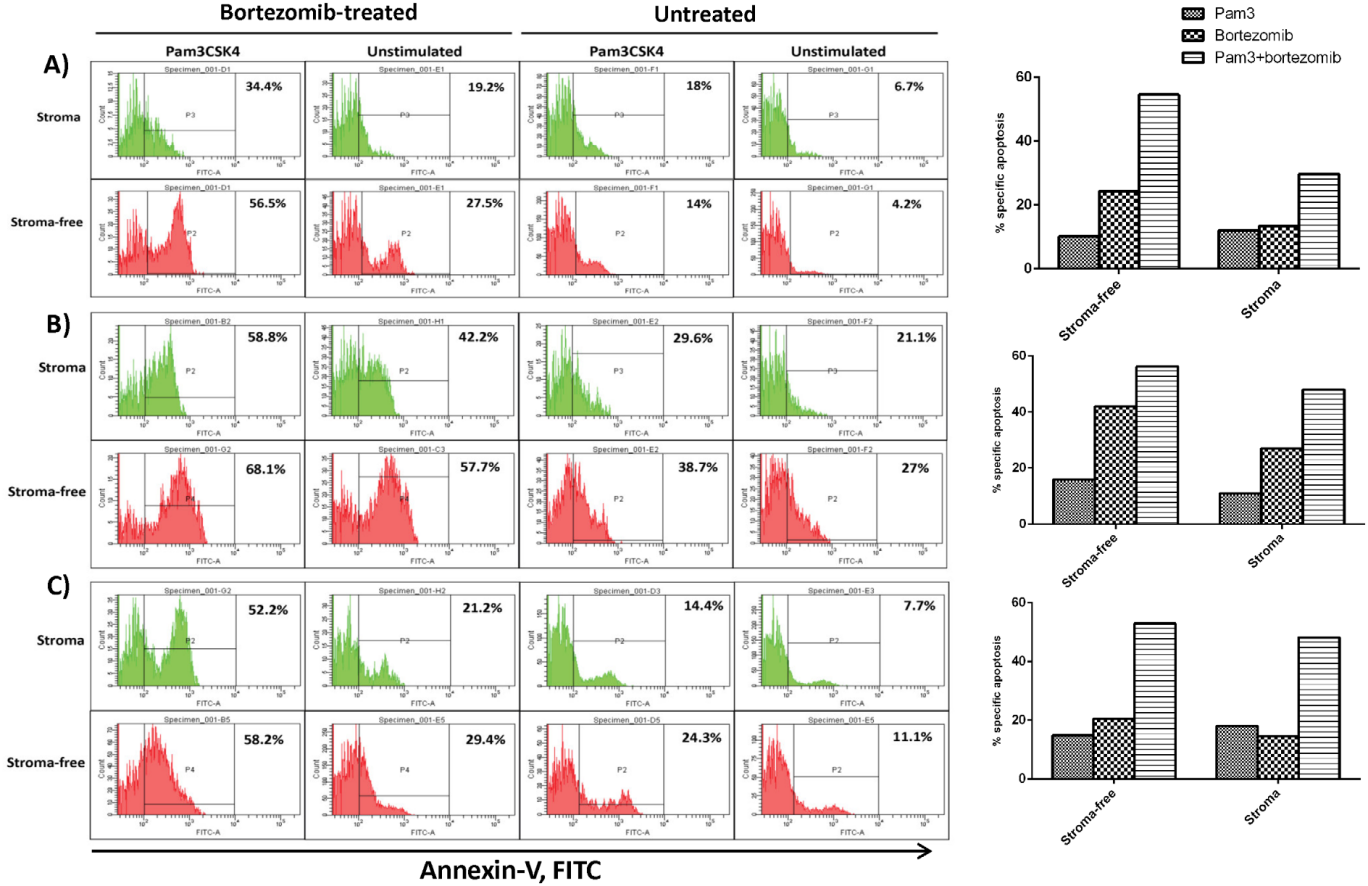
The apoptosis enhancing effect of Pam3CSK4 on HMCLs in the context of myeloma patient bone marrow stromal cells. L363 (panel **A**), OPM-2 (panel **B**) and U266 (panel **C**) were stimulated for 24 hours with Pam3CSK4, adhered to human bone marrow isolated stromal cells and then exposed to drug treatment as explained in materials and methods. Data are representative for the analysis of two MM primary BMSC samples.

Next, we explored if increased cytotoxicity to bortezomib in HMCLs after treatment with Pam3CSK4 was due to an increase in apoptosis. HMCLs were first stimulated with Pam3CSK4 for 24 hours, washed and exposed to acute bortezomib treatment and seeded onto HS-5 cells (Fig. 5) or patient BMSCs (Fig. 6), as described in materials and methods. In gated CD138^+^ cells, the percentage of annexin-V positive cells was determined to calculate specific apoptosis. As depicted in figure 5, Pam3CSK4 treatment increased the level of bortezomib-induced apoptosis in all cell lines in the presence or absence of HS-5 cells, which further confirmed the adhesion-independent effect of TLR1/2 stimulation on HMCLs viability. Of note, Pam3CSK4 itself also left a partial apoptotic effect which was more pronounced in U266 cell line. Interestingly, varying levels of cell adhesion-mediated drug resistance (CAM-DR) were detected in the context of patient primary BMSCs for all cell lines, which was reversed by the Pam3CSK4+bortezomib treatment (Fig. 6). These findings demonstrate that Pam3CSK4 increases the apoptotic effect of bortezomib in the context of BMSCs.

### Apoptosis Enhancing Effects of Pam3CSK4 on HMCLs in BMSCs Context may be Caspase Dependent

**Figure 7:**
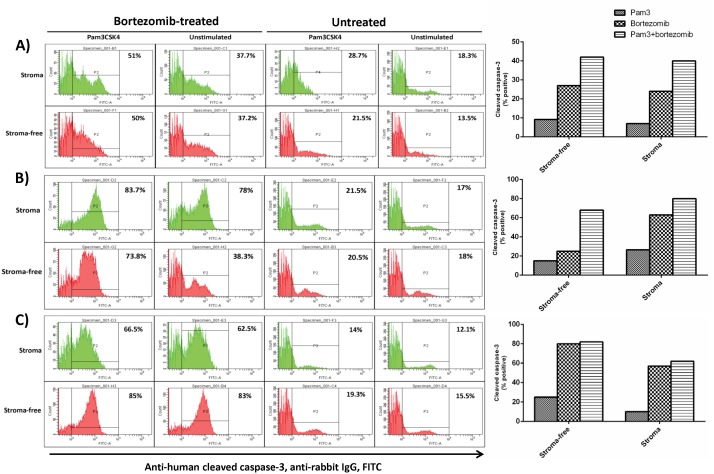
Pam3CSK4 induces apoptosis enhancing effects in bortezomib-treated HMCLs partly through caspase-3 activation. L363 (panel **A**), OPM-2 (panel **B**) and U266 (panel **C**) were stimulated for 24 hours with Pam3CSK4, adhered to primary human bone marrow isolated stromal cells and then exposed to drug treatment and cleaved caspase-3 FACS analysis, as explained in materials and methods. Pam3CSK4 alone weakly induced cleaved caspase-3 expression (compared to baseline) and its combination with bortezomib induced a higher level of cleaved caspase-3 protein in L363 and OPM-2 cell lines. Data are representative for the analysis of two MM primary BMSC samples.

Only limited details are known on the effect of TLR activation on apoptotic signaling pathways. We further investigated if Pam3CSK4 could mediate increased apoptosis via caspase-3 activation. HMCLs were first Pam3CSK4-stimulated and drug-treated as detailed in materials and methods. Using FACS analysis the percent apoptotic cells was determined after gating CD138-positive cells (Fig. 7). As expected bortezomib increased cleaved caspase-3 in all conditions for all cell lines indicating it activated caspase-3 pathway [31]. Treatment with Pam3CSK4 alone augmented the cleaved caspase-3 level to different extents in the 3 HMCLs and combination of Pam3CSK4 with bortezomib increased the level of cleaved caspase-3 mostly in an additive manner. Above findings suggest that Pam3CSK4 may contribute to apoptosis through the activation of caspase-3.

## Discussion

In the present study, we found that TLR1/2 triggering results in a heterogeneous functional response of HMCLs in terms of integrin surface expression and adhesion to BMSCs. OPM-1, OPM-2 and NCI-H929 myeloma cell lines showed a decrease in adhesion to BMSCs after TLR-1/2 activation, which was accompanied with a down-regulation in surface expression of β7 integrin. Furthermore, blocking experiments confirmed a significant contribution of β7 integrin in their adhesion, although this may not exclude the involvement of other integrins such as α4 and αVβ3 in their basal adhesion. Additionally, OPM-1, OPM-2 and NCI-H929 displayed significant increases in surface expression of α4 and αVβ3 following TLR-1/2 activation, but this upregulation appeared to have no functional effects in the adhesion to BMSCs. The integrin α4β7 has also been involved in adhesion of MM peripheral B cells to FN and BMSC [32]. Of note, the anti-β7 antibody used for blocking experiments is well known to detect only β7 epitopes regardless of its heterodimer partners [33], and thus affirms the involvement of this integrin in adhesion to BMSCs. Fravel, L363, UM-6, UM-9 and U266 HMCLs showed an increase in adhesion to BMSCs upon TLR1/2 activation. This up-regulated adhesion was demonstrated to be α4 and/or αVβ3 mediated and these two integrins are also involved in part of the baseline adhesion. It is well established that these two integrins can mediate binding of MM cells to FN and BMSCs [34], [35], and αVβ3 has been shown to be involved in invasiveness of MM cells [35], Moreover, overexpression of αVβ3 in cervical cancer has been associated with poor prognosis [36]. On the other hand, in normal human PBMCs, TLR1/2 activation increased surface expression of α4 and β7 integrins in T cells, B cells and monocytes (unpublished observation), which rather supports a common mechanism by TLR1/2 activation for α4 modulation, but also indicates a specific mechanism (possibly unique to MM cells) for β7 modulation. Preliminary experiments in siRNA-treated HMCL to knockdown MyD88 indicated the involvement of MyD88 in the TLR1/2-induced modulatory effects on expression of integrins (data not shown). Taken together, these findings imply that TLR1/2 triggering can differentially modulate α4-, αVβ3- and β7 surface expression on MM cells and α4-, αVβ3- and β7-mediated adhesion to stromal cells.

Adhesion of MM cells to BMSCs is well known to render myeloma cells resistant against cytotoxic and apoptotic signals [37], [38], [39], [40], [41]. Furthermore, adhesion-induced drug resistance is suggested to be associated with increased adhesion to fibronectin and with up-regulation of α4, thus cells with a higher expression of this integrin molecule display a drug-resistant phenotype [30]. In this study it is shown that Pam3CSK4 treatment upregulates expression of α4 in all HMCLs. However, some cell lines showed increased adhesion to BMSCs, while others showed decreased adhesion. The effect TLR1/2 activation in HMCLs on viability and drug sensitivity was further shown in three cell lines L363, OPM-2 and U266. Interestingly, Pam3CSK4 increased sensitivity (lower IC50) to bortezomib in the presence or absence of BMSCs, which was accompanied with increased apoptosis. Pam3CSK4 alone stimulated a low apoptotic effect in all HMCLs in the presence or absence of HS-5 or primary BMSCs, while combination with bortezomib induced a higher level of apoptosis. It should be noted that CAM-DR detected in some cell lines was completely eliminated by combined treatment of Pam3CSK4+bortezomib. These findings suggest that the effect of TLR1/2 stimulation on adhesion to BMSCs does not control their drug resistance or sensitivity. Thus, upregulation of adhesion to BMSCs which was shown to be mostly α4 integrin-mediated did not reduce drug-induced cell death. Likewise, downregulation of adhesion to BMSCs which was shown to be β7 integrin-mediated did not increase drug-induced cell death. A recent study demonstrated that knocking down the β7 integrin gene in MM cells decreased their adhesion to FN and BMSCs and reversed CAM-DR [33]. Additionally, blocking α4 integrin with specific antibodies increased their drug sensitivity in MM cells [30], [42], and bortezomib reversed CAM-DR in MM cells through downregulation of α4 integrin [43]. These studies support involvement of α4 and β7 integrins in controlling drug sensitivity of MM cells. However, in this study, Pam3CSK4 apparently bypassed this involvement and increased drug sensitivity of HMCLs irrespective of their adhesion pattern.

Bortezomib stimulates caspase-3 activation in MM cells, triggering apoptosis [31]. The TLR1/2 ligand (Pam3CSK4) has been shown to induce apoptosis in monocytes [44], but whether it activates caspase-3 in MM cells has not been demonstrated. Here we show that Pam3CSK4 increases the level of activated caspase-3 in HMCLs in the presence or absence of BMSCs. Combining bortezomib with Pam3CSK4 increased the level of cleaved caspase-3 in L363 and OPM-2 but not in U266. The level of cleaved caspase-3 induced by bortezomib alone paralleled CAM-DR in L363 and OPM-2 cell lines (stroma compared to stroma-free conditions) but not in U266. Although the role of caspase-3 in the induction apoptosis is well-established, other studies showed that other isoforms such as caspase-2 contributes to bortezomib-induced apoptosis in HMCL [45]. Our study also suggests that Pam3CSK4 may engage other mechanisms than caspase-3 to enhance bortezomib-induced apoptosis in HMCLs, and that inhibition of caspase signaling may only partly explain CAM-DR at least in some HMCLs. Further studies using specific inhibitors or expression-knockdown approaches should delineate the role of different caspase isoforms in TLR1/2-stimulated apoptosis in MM cells.

In summary, this study is the first to delineate the modulatory effects of TLR1/2 triggering on adhesion of HMCLs to BMSCs and identify the integrin molecules involved in this interaction. It shows that following TLR1/2 activation on HMCLs, expression of β7 integrin is downregulated, and that Pam3CSK4 increases drug sensitivity of HMCLs in the context of BMSCs. On this basis, although further research is essential, our findings suggest TLR1/2 as a potential target in MM to decrease resistance to the cytotoxic action of chemotherapeutic agents.
